# Acute Toxicity and Quality of Life in a Post-Prostatectomy Ablative Radiation Therapy (POPART) Multicentric Trial

**DOI:** 10.3390/curroncol29120733

**Published:** 2022-11-30

**Authors:** Raffaella Lucchini, Ciro Franzese, Suela Vukcaj, Giorgio Purrello, Denis Panizza, Valeria Faccenda, Stefano Andreoli, Gian Luca Poli, Davide Baldaccini, Lorenzo Lo Faro, Stefano Tomatis, Luigi Franco Cazzaniga, Marta Scorsetti, Stefano Arcangeli

**Affiliations:** 1 School of Medicine and Surgery, University of Milan Bicocca, 20126 Milan, Italy; 2 Department of Radiation Oncology, ASST Monza, 20900 Monza, Italy; 3 Department of Biomedical Sciences, Humanitas University, Via Rita Levi Montalcini 4Pieve Emanuele, 20090 Milan, Italy; 4 Department of Radiotherapy and Radiosurgery, Humanitas Clinical and Research Center—IRCCS, Rozzano, 20089 Milan, Italy; 5 Department of Radiation Oncology, ASST Papa Giovanni XXIII, 24127 Bergamo, Italy; 6 Department of Medical Physics, ASST Monza, 20900 Monza, Italy; 7 Department of Physics, University of Milan, 20122 Milan, Italy; 8 Department of Medical Physics, ASST Papa Giovanni XXIII, 24127 Bergamo, Italy

**Keywords:** prostate cancer, postoperative setting, SBRT

## Abstract

Background: The aim of this study was to investigate the feasibility of ultrahypofractionated radiotherapy to the prostate bed in patients with biochemical and/or clinical relapse following radical prostatectomy who were enrolled in the prospective, observational, multicentric POPART trial (NCT04831970). Methods: Patients with post-radical prostatectomy PSA levels of ≥0.1–2.0 ng/mL and/or local relapse at PSMA PET/CT or multiparametric MRI were treated with Linac-based SBRT on the prostate bed up to a total dose of 32.5 Gy in five fractions every other day (EQD2_1.5_ = 74.2 Gy). Maximum acute toxicity was assessed using the Common Terminology Criteria for Adverse Events version 5 scale. International Consultation on Incontinence Questionnaire—Short Form (ICIQ-SF) and Prostate Cancer Index Composite for Clinical Practice (EPIC-CP) scores were assessed at baseline and during the follow-up. Results: From April 2021 to June 2022, thirty men with a median age of 72 years (range 55–82) were enrolled in three centers. The median PSA level before RT was 0.30 ng/mL (range 0.18–1.89 ng/mL). At 3 months post-treatment, no GI or ≥2 GU side effects were reported; three patients (10%) experienced Grade 1 GU toxicity. No changes in ICIQ-SF or in the urinary domains of EPIC-CP were observed, while a transient worsening was registered in the bowel domain. At the same time point, all but two patients, who progressed distantly, were found to be biochemically controlled with a median post-treatment PSA level of 0.07 ng/mL (range 0–0.48 ng/mL). Conclusions: Our preliminary findings show that SBRT can be safely extended to the postoperative setting, without an increase in short-term toxicity or a significant decline in QoL. Long-term results are needed to confirm this strategy.

## 1. Introduction

Regardless of the two settings (adjuvant or salvage), external-beam radiation therapy (RT) for prostate cancer is usually a protracted course, since a total dose of 64 Gy to 72 Gy is needed to be effective [1,2]. In addition, a randomized trial [3] has recently proven the benefit of extending salvage RT to the pelvic lymph nodes in combination with short-term androgen deprivation therapy (ADT) in patients with a detectable or rising prostate-specific antigen (PSA) level after prostatectomy. All these strategies share a typical rate of 1.8 Gy to 2.0 Gy per treatment, taking up to 39 fractions over the course of 8 weeks to be completed, which is extremely time-consuming and barely convenient for both patients and the healthcare system.

Since the end of the 1990s, dose–response analyses of patients with prostate cancer treated with both external-beam RT and brachytherapy have led to the assumption that the α/β ratio of prostate cancer is lower than that for most other tumors and approaches a value characteristic of late-responding tissues. Values between 1.2 and 3.9 Gy have been proposed [4,5,6]. Therefore, delivering the same equivalent dose at 2 Gy per fraction (EQD2) to the prostate using a larger than conventional (2 Gy) fraction size would not affect late side effects and would result in a sparing effect on early responding normal tissues. Hypofractionation would thus reduce early side effects (if overall treatment time is left constant) or could be used to shorten overall treatment time owing to the increased therapeutic index. This strategy is expected to be at least isoeffective in terms of tumor control but with the associated advantages of cost, logistics and patient convenience. However, while non-inferiority trials [7,8,9,10] have confirmed these premises and validated moderate hypofractionation as an established treatment modality in the radical setting, such an approach has traditionally been hampered in the postoperative setting, likely due to the concerns that high radiation doses in the anastomosis (where most recurrences occur) may lead to tissue injury. To date, few studies [11,12] have explored the use of hypofractionation for salvage RT in patients with biochemical recurrence after prostatectomy, showing excellent outcomes in terms of both efficacy and toxicity. However, differences in follow-up, radiation techniques and treatment schedules prevent any definitive conclusion. Data are even more scarce for Stereotactic Body Radiation Therapy (SBRT), which is on the shortest end of the hypofractionation spectrum, with only a phase II trial reporting on the Quality-of-Life Outcomes and Toxicity Profile of 100 patients who received post-prostatectomy SBRT doses of 30–34 Gy in five fractions to the prostate bed, either with MRI-guided RT or standard computed tomography-guided RT [13].

In this study, we aimed to report on the short-term physician-scored genitourinary (GU) and gastrointestinal (GI) toxicities and the Quality of Life (QoL) of a cohort of patients enrolled in a post-prostatectomy SBRT multicentric trial.

## 2. Methods

### 2.1. Study Design

The POPART trial was a multicentric, prospective, observational trial (NCT04831970) aimed at evaluating the feasibility of postoperative SBRT for prostate cancer in terms of toxicity and QoL. The study was approved by the Ethical Committees of the participating centers. All participants provided written informed consent prior to trial enrollment in agreement with the Declaration of Helsinki [14].

### 2.2. Eligibility

Patients eligible for this study must have had adenocarcinoma of the prostate treated with radical prostatectomy (any type of radical prostatectomy was permitted, including retropubic, perineal, laparoscopic or robotically assisted; there was no time limit for the date of radical prostatectomy). Additional factors were required as inclusion criteria, such as a post-radical prostatectomy PSA level between 0.1 and 2.0 ng/mL; adverse pathologic features (pathologic T3/T4 disease with or without positive surgical margins) and/or a rising prostate-specific antigen (PSA) level > 0.1 ng/mL on at least two consecutive measurements; and no distant metastases on [^18^F]-PSMA positron emission tomography (PET) within 60 days prior to registration. ADT was allowed, and its prescription was left at the physician’s discretion.

### 2.3. Treatment Planning and Radiation Delivery

The patients were immobilized in the supine position using the FeetFix (CIVCO Medical Solutions, Coralville, IA, USA) system anchored to the couch for ankle fixation, with their arms placed over their chest. To assess anatomical reproducibility and organ motion mitigation, before simulation and each treatment, the patients were administered a micro-enema and asked to fill their bladder by drinking 500 mL of still water.

The clinical target volume (CTV) was delineated according to the Groupe Francophone de Radiothérapie Urologique (GFRU) Guideline [15]. The planning target volume (PTV) included CTV with a 5 mm isotropic 3D margin, except for at the rectum interface, where the margin was kept at 3 mm. Volumetric Modulated Arc Therapy (VMAT) treatment consisted of two 6 MV or 10 MV flattening filter free (FFF) full arcs optimized to ensure that the 95% isodose covered at least 95% of the PTV. SBRT was scheduled in 5 fractions every other day for a total dose of either 31 Gy or 32.5 Gy, according to the adjuvant or salvage intent. The corresponding EQD2 considering an α/β ratio of 1.5 Gy was 68.2 Gy and 74.3 Gy, respectively. Mandatory dose–volume constraints were defined for both target coverage and the avoidance of normal adjacent tissues, including the rectum, rectum wall, bladder, bladder wall and penile bulb, as shown in Table 1. Accurate patient setup was obtained using kilovoltage cone-beam CT (CBCT) before each session to check the anatomical reproducibility.

### 2.4. Toxicity and Quality of Life Assessment

Toxicity, as defined by the National Cancer Institute Common Terminology Criteria for Adverse Events v.5.0, was assessed at baseline, at the end of treatment and every 3 months thereafter. The ICIQ-SF and the Expanded Prostate Cancer Index Composite for Clinical Practice (EPIC-CP) bowel and urinary QoL [16,17] scores were collected once prior to treatment and thereafter at the aforementioned time points via questionnaires. Paired t-test and the Wilcoxon signed-rank test were used to compare pretreatment and post-treatment EPIC-CP domain scores. The incidence of acute treatment-related GU and GI toxicities, patient QoL and PSA outcomes were computed from the start of the treatment to the last follow-up.

## 3. Results

### 3.1. Population Characteristics

From April 2021 to 30 June 2022, patients (median age, 72 years; range, 55–82) were enrolled in the multicentric POPART trial and completed the study treatment. Their baseline demographic and clinical characteristics, as well as treatment parameters, are shown in Table 2. The majority (74%) had a biochemical relapse only, while eight patients (26%) also had a local relapse. The median PSA level before RT was 0.30 ng/mL (range 0.18–1.89 ng/mL). Four patients (13%) received ADT. At baseline, the median ICIQ-SF score was 1 (range 0–8). The median PTV was 72 cc (range 14.8–250.2 cc).

### 3.2. Treatment Outcome

All patients completed the treatment according to the protocol’s schedule. After SBRT completion, only one instance of Grade 2 acute GI toxicity was documented; no ≥ Grade 2 acute GU toxicity was observed, and three patients experienced Grade 1 GU side effects. At the three-month follow-up, no GI or ≥Grade 2 GU side effects were reported; Grade 1 GU toxicity was detected in three patients (10%) (Table 3). Three months after SBRT, all but two patients, who progressed in new distant sites, were found to be biochemically controlled with a median post-treatment PSA level of 0.07 ng/mL (range 0–0.48 ng/mL).

### 3.3. Quality of Life

The ICIQ-SF assessed at baseline and 3 months after treatment remained unchanged with a median score of 1 (range 1–8). In agreement with the results of the ICIQ-SF, there was no significant decline in the median EPIC-CP scores in the urinary domains at the end of the treatment and 3 months after. Conversely, in the bowel domain, a transient worsening was observed at the end of SBRT with a median value of 1.8 (±0.2) from the baseline of 0.7 (±0.1), but this returned to the pre-treatment level at a later time point, with a median score of 0.8 (±0.1) at 3 months (Table 4).

## 4. Discussion

It has been established that, in the salvage setting, for each additional Gy, there is an approximately 2.5% improvement in 5 y biochemical relapse-free survival (bRFS) [18]. However, two phase III dose-escalation studies using conventional schedules showed only increased rates of GI side effects without providing any benefits to the patients [1,2]. Due to the low α/β ratio of prostate cancer, hypofractionation might represent a window of opportunity aimed at maintaining the same local control (isoeffective) while potentially decreasing the risk of treatment-related toxicities, as already proven in the primary setting [7,8,9,10]. Historically, in the postoperative setting, the use of hypofractionation has for a long time been discouraged because microscopic relapse in the prostate bed can only be inferred because of some concerns that high radiation doses absorbed by tissues, which have been already injured by surgery, could have resulted in an increased risk of developing major toxicities. Indeed, some evidence became available from a number of retrospective studies of salvage moderately hypofractionated RT with small sample sizes and different endpoints. Their results were summarized in a systematic review [11], involving more than 1200 patients, which showed that an EQD2 dose > 70 Gy was associated with better bRFS (namely, 83%, 85.4% and 100% in three studies) and a 5-year ≥ Grade 2 toxicity ranging between 7.3% and 18.1%. Another metanalysis [12] on five retrospective studies of moderate hypofractionation in the salvage setting in 369 patients reported encouraging results of 3-year bRFS of 73% and late ≥ Grade 2 GU and GI toxicities of 6% and 3%, respectively. However, differences in the number of patients, fractionation schedules and the duration of follow-up raise some uncertainties and lower the quality of the evidence. More robust data came from a phase II single trial [19] reporting on 61 patients treated with a salvage hypofractionated regimen of 15 fractions of 3.4 Gy each: with a median follow-up of 16 months, only two cases of acute (primary endpoint) and late > Grade 3 GU events were documented, along with bRFS rates of 95.1%. When approaching extreme hypofractionation, the latest evidence was provided by the largest prospective study of post-prostatectomy SBRT [13] reporting on 100 participants treated with a median prostate bed dose of 32 Gy in five fractions: at a median follow-up of 29.5 months, acute and late Grade 2 GU toxicities were both 9%, while acute and late Grade 2 GI toxicities were 5% and 0%, respectively. Interestingly, those treated with MRI-guided RT (MRgRT) showed a 30% reduction in any grade acute GI toxicity and improved bowel QoL.

Our prospective study is among the few reporting on early toxicity and QoL assessment following postoperative SBRT. Besides the Scimitar trial [13], only a metanalysis [20] is available on extreme hypofractionation in the salvage setting, including 11 retrospective series, which showed acceptable rates of acute and late GU and GI toxicity; however, in all but two, the radiation target was a macroscopic recurrence and not the prostate bed. When our study was conceived, we designed two slightly different SBRT regimens according to the adjuvant or salvage setting. As a matter of fact, all patients received salvage SBRT for biochemical recurrence, and none were treated on the basis of negative prognostic findings at pathologic specimen examination with PSA controlled. This attitude likely acknowledged the results from the Artistic metanalysis [21], which suggested that early salvage RT is the preferable treatment policy, as it can spare many patients from the overtreatment of upfront RT and its associated adverse events. Likewise, ADT was left to the physician’s discretion because, at that time, there was no compelling evidence that it added clear benefit in the salvage setting. Recently, a randomized trial [3] for the first time showed that the combination of short-term ADT with salvage RT extended to treat the pelvic lymph nodes led to significant reductions in progression in patients with a detectable or rising PSA after prostatectomy. This benefit however came at the cost of a significant increase in the risk of late ≥ Grade 2 blood or bone marrow events (*p* = 0.0060), attributable to the addition of pelvic nodal RT. In view of the detrimental prognosis of such hematologic toxicity, namely, leukopenia, associated with extended-field RT [22,23], hypofractionation to the prostate bed may only result in providing a protective effect against leukotoxicity [24], thus increasing its therapeutic gain. 

In our series, the rates of clinically relevant acute and subacute side effects and QoL were almost negligible and nearly equivalent to those reported in the retrospective series employing moderate hypofractionation, mostly with IMRT [11,12,25,26,27]. These results also compare favorably to the acute ≥ Grade 2 GU toxicity of 0–8% and the ≥Grade 2 GI toxicity of 33–58% observed in patients receiving similar doses on two prior phase I SBRT trials [28,29] and with two phase II trials using either moderate hypofractionation [19] or extreme hypofractionation [13]. Notably, in the latter study, the authors attributed the improvements in GI toxicity and QoL to the narrower PTV margins obtained with MRgRT (3 mm) compared to the 5 mm used with standard computed tomography-guided RT (CTgRT). In our study, treating the prostate bed with a similar schedule on a Linac platform and using an anisotropic expansion for PTV of 5 mm in each direction, except for at the rectum interface (3 mm), resulted in a single instance of acute Grade 2 GI toxicity. Although the real-time tracking of the anterior rectal wall was not an option, as for MRgRT, the PTV margin’s drop to 3 mm posteriorly was still considered safe due to the short beam-on time enabled by the flattering filter free (FFF) modality, as well as the intrafraction motion mitigation protocol obtained by a strict bowel and bladder set-up, which ensured target stabilization and anatomical reproducibility. Furthermore, the use of [^18^F]-PSMA PET before treatment excluded distant metastases, even at low PSA levels, thus aiding patient selection and accordingly improving oncologic outcomes, as already elucidated in the Empire 1 phase II/III trial [30], where patients whose treatment was guided by another form of advanced imaging (^18^F-Fluciclovine PET/CT) exhibited a remarkable benefit in 5-year bRFS. 

## 5. Conclusions

Despite the follow-up being too short to consider our SBRT schedule safe in the long term, we believe that our findings are encouraging, at least at early time points, and that they highlight that highly focused radiation in a few fractions to the prostate bed with robust conformality and modulation, abrupt dose fall off and image guidance can be safely extended to the postoperative setting, thus broadening the attractiveness of extreme hypofractionation and enhancing its already unmatched cost-effectiveness profile.

## Figures and Tables

**Table 1:** Treatment Planning Dose–Volume Constraints for Post-prostatectomy SBRT.

PTV D95%	D95% ≥ 95%	≥30.875 Gy
PTV Maximum	Dmax < 107%	<34.800 Gy
Rectal Wall Maximum	Dmax < 107%	<34.800 Gy
Rectal Wall D (1 cc) (Dose to 1 cc)	<100% of Prescribed Dose	<32.500 Gy
Rectal wall D50% (Dose to 50% Volume)		<22.500 Gy
Bladder Wall Maximum	Dmax < 107%	<34.800 Gy
Bladder Wall D (10 cc) (Dose to 10 cc)	<100% of Prescribed Dose	<32.500 Gy
Bladder Wall D25% (Dose to 25% Volume)	<100% of Prescribed Dose	<32.500 Gy
Bladder Wall D50% (Dose to 50% Volume)		<24.000 Gy
Small/Large Bowel Maximum		<20.000/25.000 Gy
Penile Bulb	<100% of Prescribed Dose	3 cc 24.000 Gy
Femur Maximum		<30.000 Gy

**Table 2:** Patients, Disease and Treatment Characteristics.

Age	
Median	72 [range 55–82]
PSA pre-prostatectomy (ng/mL)	
Median	6.04 [range 3.30–17.25]
Gleason score	
6 (3 + 3)	4 (13%)
7 (3 + 4)	12 (40%)
7 (4 + 3)	11 (37%)
8 (4 + 4)	2 (6.5%)
9 (4 + 5)	1 (3.5%)
ISUP Grade Group	
1	4 (13%)
2	12 (40%)
3	11 (37%)
4	2 (6.5%)
5	1 (3.5%)
Pathological T stage	
pT2	19 (64%)
pT3a	8 (26%)
pT3b	3 (10%)
Positive Margins	
R1	11 (37%)
R1 and positive apex	8 (26%)
Time from prostatectomy (months)	
Median	54.5 [range 7–155]
Clinical relapse	
Yes	8 (26%)
No	22 (74%)
PSA pre-SBRT (ng/mL)	
Median	0.30 [range 0.18–1.89]
ADT	
Yes	4 (13%)
No	26 (87%)
CTV (cc)	
Median	29.39 [range 4.40–149.00]
PTV (cc)	
Median	72 [range 14.8–250.2]

**Table 3:** Maximum acute toxicity during radiation or within 3 months after RT.

	0	1	2	≥3
GI	29 (97%)	—	1 (3%)	—
GU	27 (90%)	3 (10%)	—	—

**Table 4:** Patients reported HRQOL using EPIC-CP.

		Mean ± SD Score	
	Baseline	End of Treatment	3 Months
Urinary incontinence	1.6 ± 0.1	1.8 ± 0.1	1.6 ± 0.1
Urinary irritation/obstruction	1.2 ± 0.1	1.8 ± 0.2	1.6 ± 0.1
Bowel symptoms	0.7 ± 0.1	1.8 ± 0.2	0.8 ± 0.1
Sexual dysfunctions	5.3 ± 0.2	5.3 ± 0.2	5.8 ± 0.2
Hormonal symptoms	1.1 ± 0.1	1.3 ± 0.1	1.6 ± 0.1

## Data Availability

The datasets supporting the conclusions of this article are included within the article.
